# Comparative genomic analysis of *Ralstonia solanacearum* reveals candidate genes for host specificity

**DOI:** 10.1186/s12864-015-1474-8

**Published:** 2015-04-08

**Authors:** Florent Ailloud, Tiffany Lowe, Gilles Cellier, David Roche, Caitilyn Allen, Philippe Prior

**Affiliations:** CIRAD, UMR PVBMT, F-97410 Saint-Pierre, La Réunion France; Department of Plant Pathology, University of Wisconsin-Madison, Madison, WI USA; Anses - Plant Health Laboratory, F-97410 Saint-Pierre, La Réunion France; Institut de Génomique, Genoscope, Commissariat à l’Energie Atomique (CEA), Evry, Paris, France; Département de Santé des Plantes et Environnement, (SPE) Inra, Paris, France

**Keywords:** *Ralstonia solanacearum*, Comparative genomics, Host adaptation

## Abstract

**Background:**

*Ralstonia solanacearum* is a vascular soil-borne plant pathogen with an unusually broad host range. This economically destructive and globally distributed bacterium has thousands of distinct lineages within a heterogeneous and taxonomically disputed species complex. Some lineages include highly host-adapted strains (ecotypes), such as the banana Moko disease-causing strains, the cold-tolerant potato brown rot strains (also known as R3bv2) and the recently emerged Not Pathogenic to Banana (NPB) strains.

**Results:**

These distinct ecotypes offer a robust model to study host adaptation and the emergence of ecotypes because the polyphyletic Moko strains include lineages that are phylogenetically close to the monophyletic brown rot and NPB strains. Draft genomes of eight new strains belonging to these three model ecotypes were produced to complement the eleven publicly available *R. solanacearum* genomes. Using a suite of bioinformatics methods, we searched for genetic and evolutionary features that distinguish ecotypes and propose specific hypotheses concerning mechanisms of host adaptation in the *R. solanacearum* species complex. Genome-wide, few differences were identified, but gene loss events, non-synonymous polymorphisms, and horizontal gene transfer were identified among type III effectors and were associated with host range differences.

**Conclusions:**

This extensive comparative genomics analysis uncovered relatively few divergent features among closely related strains with contrasting biological characteristics; however, several virulence factors were associated with the emergence of Moko, NPB and brown rot and could explain host adaptation.

**Electronic supplementary material:**

The online version of this article (doi:10.1186/s12864-015-1474-8) contains supplementary material, which is available to authorized users.

## Background

Although many plant pathogens are narrowly adapted to one or a few related plant hosts, *Ralstonia solanacearum* has an unusually broad host range that includes monocotyledonous and dicotyledonous hosts [1]. *R. solanacearum* infects plant vascular systems, causing diverse bacterial wilt diseases. Its extensive host range, together with a wide geographic distribution, makes it one of the world’s most destructive crop pathogens [2]. *R. solanacearum* has many distinct lineages within a heterogeneous and taxonomically disputed species complex. Extensive phylogenetic analysis demonstrates that the species complex contains four phylotypes that correspond to geographic origin; phylotype I strains originated in Asia, phylotype II strains came from the Americas, phylotype III strains originated in Africa, and phylotype IV strains are from the Indonesian archipelago [3]. Within each phylotype, strains can be further subclassified into sequevars based on the similarity of a 750-bp fragment of the endoglucases (*egl)* gene [3]. *R. solanacearum* strains share a conserved core genome that is presumably essential for their common biology: colonizing plant xylem vessels and causing wilt symptoms [4,5]. However, the group’s pan-genome contains over 16,000 coding sequences (CDSs), and individual strains in the species complex vary enormously with respect to both epidemiology and host range. For example, the *R. solanacearum* species complex includes strains adapted to eucalyptus, mulberry, clove, ginger, banana, peanut, and solanaceous plants [1].

Functional genetic analyses have identified many of *R. solanacearum’s* broadly conserved common virulence mechanisms [6,7]. However, the molecular processes that are responsible for strains’ adaptation to particular hosts are not well understood. A few studies have identified specific type III effectors and metabolic traits associated with host range [8-11]. However, these findings were validated in only a few model strains using plants that fail to represent the large diversity of the species complex’s host range. Previous comparative analyses did not identify strong host specificity factors but were based on strains with a limited genetic diversity or failed to consider the entire host spectrum of each strain [12,13]. A recent study that characterized the bacterium’s genetic and phenotypic heterogeneity allowed us to select new model strains and to further explore the underlying mechanisms that determine host range [14].

Microarray and multilocus sequence typing (MLST) analyses and surveys of biological diversity demonstrated the existence of several clonal lineages adapted to specific host plants inside the American phylotype II group of the *R. solanacearum* species complex [12,14-16]. Lineages consisting of strains with similar host ranges are grouped into ecotypes, as they represent genetically distinct populations adapted to particular ecological niches within the *R. solanacearum species complex* (RSSC). These lineages include the banana Moko disease-causing strains, the cold-tolerant potato brown rot strains (historically and for regulatory purposes known as Race 3, biovar 2 or R3bv2) and the Not Pathogenic to Banana (NPB) strains, a recently emerged group that does not wilt banana despite its phylogenetic location in sequevar 4; all other sequevar 4 strains cause Moko disease. We hypothesized that host range determinants could be identified by comparing the genomes of closely related strains that have highly divergent host ranges. The polyphyletic nature of the Moko ecotype and the unexpectedly close relationship of some Moko lineages to the monophyletic brown rot and NPB ecotypes make these highly adapted strains a robust model for the study of host adaptation.

In this study, we sequenced a representative group of strains from each of these ecotypes and analyzed their genomes using multiple comparative genomics methods. Genomes were compared using phyletic profiling to determine ecotype-specific gene content. We identified genetic variations associated with NPB and brown rot strains emergence. To gain a better understanding of the polyphyly of Moko strains, horizontal gene transfers (HGTs) were also investigated using an explicit phylogenetic method. Each of these analyses has proven successful in several other plant pathogenic bacteria, including *Pseudomonas syringae* [17] and *Xanthomonas* sp. [18,19], and has provided evidence that specific gene content, genetic variations and HGT can explain host adaptation.

These comparative genomic analyses identified several virulence factors associated with NPB and brown rot strain emergence, along with Moko strain polyphyly. However, there were surprisingly few differences in lineage-specific gene content that could explain the host adaptation of the various lineages despite the high plasticity of the pan-genome.

## Results

### Host-adapted strains from phylotype II offer a model to study host adaptation

The general features of the *R. solanacearum* genomes analyzed in this study are presented in Table 1. We selected eight phylotype II strains for sequencing based on their sequevar classifications and on phenotypic data that indicated host adaptation. Eleven additional RSSC genomes were obtained from public databases. The three brown rot strains form a monophyletic group adapted to temperate climate; these sequevar 1 strains are typically isolated from potato in the highland tropics. The Moko disease strains form a polyphyletic group adapted to banana and are represented in this analysis by seven strains from sequevars 3, 4, 6 and 24, all isolated from plants in the genus *Musa*. The NPB strains form an emerging monophyletic group that appears to have diverged recently from *Musa*-infecting strains in sequevar 4. This group is represented by two strains, one isolated from *Cucumis* and one from *Heliconia* [15].

**Table 1:** **General characteristics of the strains and genomes used in this study**

**Phylotype - sequevar**	**Strain**	**Genome length (Mb)**	**GC %**	**#Contig**	**#CDS**	**Isolated from**	**Geographic origin**
I	GMI1000	5.81	66.9%	2	5635	Tomato	Guyana
IIB-1	IPO1609	5.24	66.7%	102	5203	Potato	Netherlands
	UW551	5.22	64.8%	561	5301	Geranium	Kenya
	UW491	5.27	66.7%	222	5035	Potato	Colombia
IIB-3	MolK2	5.48	66.7%	30	5438	Banana	Philippines
	CFBP1416*	5.68	66.6%	653	5722	Plantain	Costa Rica
	CIP417*	5.47	66.8%	609	5398	Banana	Philippines
IIB-4	UW179*	5.37	66.7%	590	5354	Banana	N/A
	UW163*	5.48	66.6%	572	5467	Plantain	Peru
	Po82	5.43	66.6%	115	5019	Potato	Mexico
IIB-4 NPB	CFBP6783*	5.54	66.7%	655	5505	*Heliconia*	French West Indies
	IBSBF1503*	5.45	66.7%	633	5452	Cucumber	Peru
IIA-7	K60	5.33	66.7%	23	5102	Tomato	United States
IIA-6	Grenada91*	5.41	66.6%	670	5365	Banana	Grenada
IIA-24	B50*	5.49	66.4%	1088	5648	Banana	Peru
III	CMR15	5.61	65.0%	3	5149	Tomato	Cameroon
IV	PSI07	5.62	64.5%	3	5247	Tomato	Indonesia
	BDB R229	5.23	66.3%	13	5051	Banana	Indonesia
	*R. syzygii* R24	5.45	65.9%	2	5239	Clove	Indonesia

* Genomes sequenced during this study.

**Figure 1:**
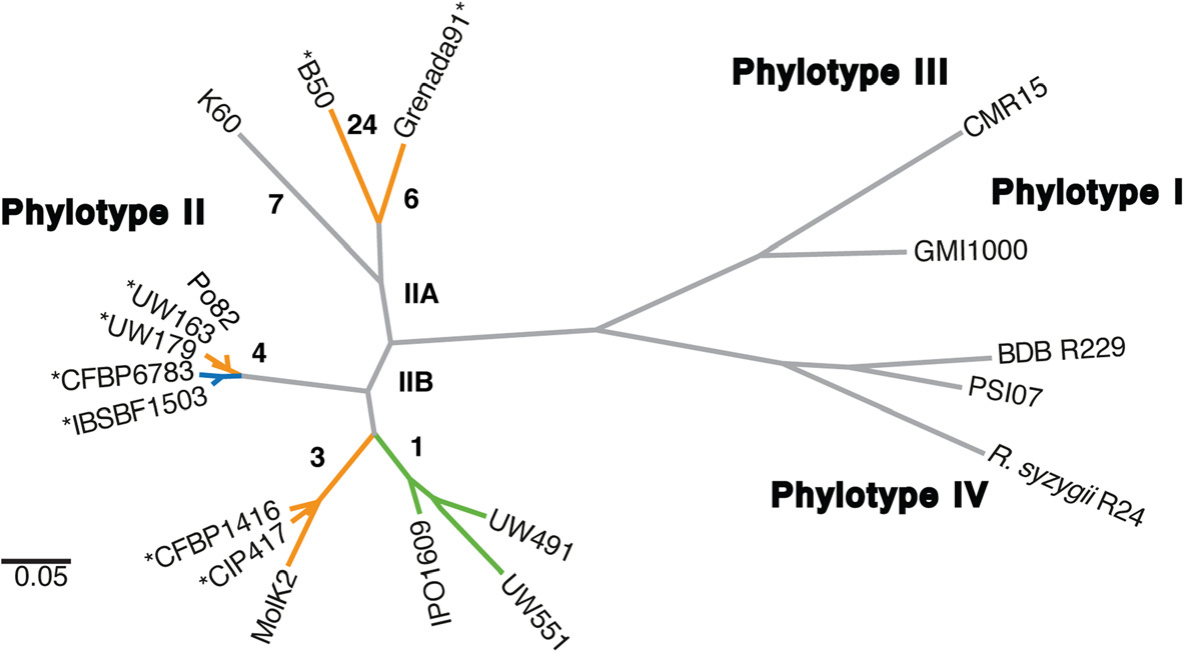
**Phylogenetic tree of the** ***R. solanacearum*** **species complex inferred from whole-genome comparisons.** The tree was computed from MUMi genomic distances. Stars next to strain names indicate genomes sequenced during this study. Phylotype and sequevars are given along branches. Colors indicate ecotypes. Orange: Moko (banana) strains; Blue: NPB strains (Not Pathogenic to Banana), Green: Potato brown rot strains. The scale bar corresponds to MUMi distances.

We determined draft sequences of a total of eight new phylotype II draft genomes. The resulting whole-genome data were used to confirm the phylogenetic relationships previously inferred from the phylotype-sequevar classification. We used Maximum Unique Matches index (MUMi) genomic distances [20] to generate a new, more complete phylogenetic tree of the *R. solanacearum* species complex (Figure 1). The tree exhibits a similar topology as the previously inferred phylogeny, which was based on microarray or MLST data. Although the strains in phylotype II are closely related to the relative context of the species complex, the Moko group is clearly polyphyletic and includes four distinct lineages that correspond to sequevars 3, 4, 6 and 24. Interestingly, Moko sequevar 3 is closely related to the monophyletic potato brown rot lineage, and Moko sequevar 4 is even more closely related to the monophyletic NPB lineage.

**Figure 2:**
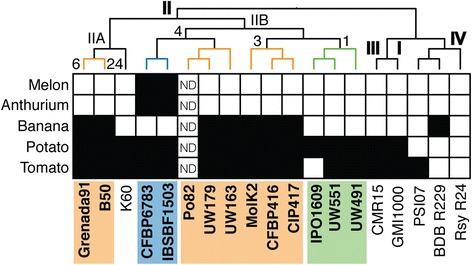
**Host range of sequenced** ***R. solanacearum*** **strains.** Black squares indicate compatible interactions, and white squares indicate incompatible interactions. The tree located alongside the matrix is the same as the one depicted in Figure 1. Colors indicate ecotypes. Orange: Moko (banana) strains; Blue: NPB strains (Not Pathogenic to Banana), Green: Potato brown rot strains.

To confirm the host-adapted nature of the selected phylotype II strains, we initially conducted pathogenicity assays under tropical conditions using tomato, potato, banana (*Musa*), melon (*Cucumis*) and *Anthurium* as representative hosts. The pathogenicity profiles obtained from these assays clearly demonstrate the host adaptation of each group (Figure 2). Moko strains from sequevars 3, 4, 6 and 24 wilted banana plants, but the sequevar 4-NPB strains did not. The NPB strains were the only group able to infect melon and *Anthurium* plants. Finally, Moko, NPB, and brown rot strains can all infect potato plants at warm temperatures, but only brown rot strains are highly virulent under cooler temperate conditions [14]. It is important to note that not all strains in phylotype II exhibit this degree of host adaptation. For example, and as expected from the literature, the broad host range of *R. solanacearum* type strain K60 (which also causes disease in tobacco, eggplant, and pepper) did not extend to banana, melon or anthurium under our experimental conditions.

### Genomic diversity of phylotype II and the *R. solanacearum* species complex

**Figure 3:**
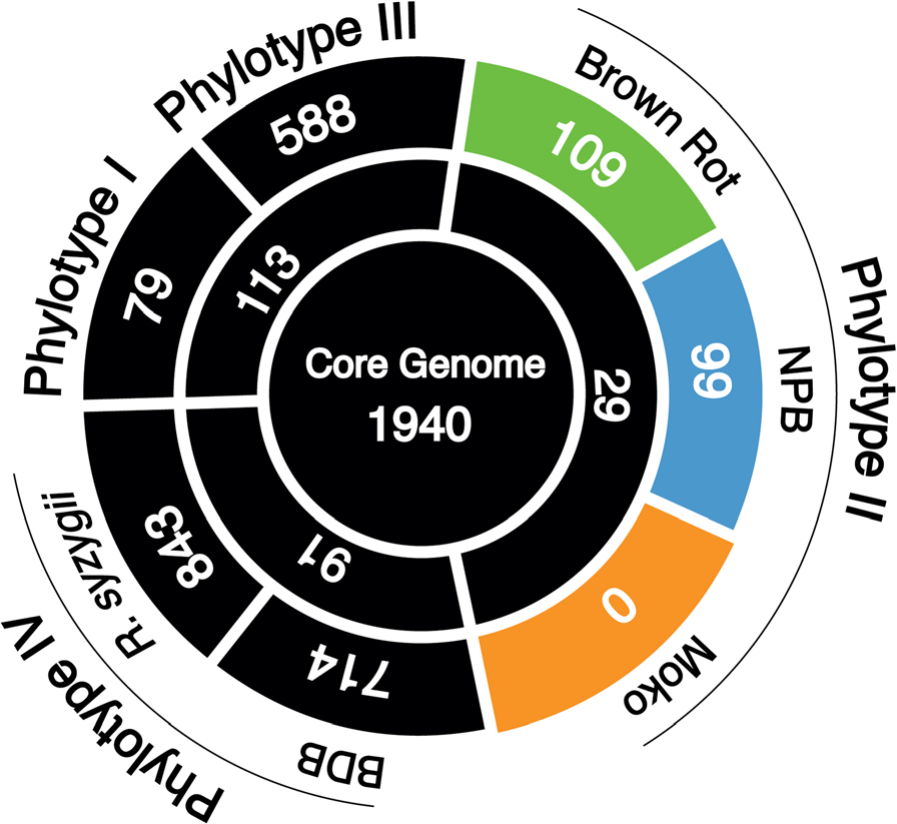
**Comparative diagram of the specific gene contents in representative groups of the species complex.** The number of genes unique to each group was determined using homolog prediction inferred with the OMA algorithm. Colors indicate ecotypes. Orange: Moko (banana) strains; Blue: NPB strains (Not Pathogenic to Banana), Green: Potato brown rot strains.

A total of 16,757 distinct homolog families were identified across the 19 *R. solanacearum* genomes; we consider these homologs to be the current pan-genome of the species complex. Compared with a previous genomic analysis of eight *R. solanacearum* genomes [5], our results expand the pan-genome size by ~70%. The inclusion of previously unsequenced and phylogenetically distinct lineages, notably sequevars 4, 6 and 24, explains the steep increase in the pan-genome size. The 1,940 loci conserved in all strains constitute the current *R. solanacearum* core genome, which represents 17% of the pan-genome and 35% of an average *R. solanacearum* genome, which contains ~5,500 CDSs. Ecotype-specific gene content was determined by individually comparing each ecotype to every other sequenced genome. The NPB and brown rot strains each exhibited only a few unique genes specific to their phylogenetic groups: 99 and 109*,* respectively (Figure 3). The Moko strains are unlikely to exhibit specific genes because this polyphyletic group is composed of distant lineages. Three hypotheses can explain the emergence of a polyphyletic Moko ecotype. First, the most common recent ancestor (MRCA) of phylotype II may have been adapted to bananas. During the subsequent clonal expansion, variations in environmental selection pressures may have promoted maintenance of the ancient phenotype in certain lineages (which still cause Moko disease) and promoted its loss in others. Alternatively, because *R. solanacearum* is capable of natural transformation [21], pathogenicity to banana may have originated in an isolated lineage after divergence from the MRCA. Subsequently, the banana-infecting trait(s) may have spread to multiple phylotype II lineages via HGT, resulting in the current polyphyletic Moko group. A third possibility is that multiple lineages underwent convergent evolution towards the banana-infecting trait, which is consistent with the absence of ecotype-specific genes.

We selected a subset of 227 known or likely virulence factors from the pan-genome based on the literature and manually annotated them (Additional file 1). These virulence factors have a broad range of functions during pathogenesis, including the secretion and synthesis of type III effectors (T3es), motility, chemotaxis, synthesis of exopolysaccharide (EPS), and degradation of plant cell walls. Compared with the other virulence factors, the T3e repertoire of *R. solanacearum* exhibits high plasticity. The pan-effectome of the species complex contains 113 T3es, but of those, only 14 effectors are present in every sequenced strain. These 14 effectors form the core effectome: RipG5, RipB, RipW, RipAC, RipAB, RipR, RipE1, RipAM, RipAN, RipAY, RipAJ, RipF1, RipAI and a PopC-like effector. The core effectome represents a ~36% size decrease relative to the previously inferred core effectome based on 11 strains [13]. On average, 64 T3es were present per strain. A total of 40 T3es were common to all Moko strains, whereas 76 were common to NPB strains, and 52 were common to brown rot strains.

Due to the large pan-genome, each strain appeared to exhibit highly diverse genetic content even within the Moko, NPB and brown rot groups. This variability can be partially explained by the estimation of gene gain and loss rates along lineages. Indeed, each phylogenetic node of the species complex appears to have undergone hundreds of gain and loss events, thus creating mosaic genomes (Additional file 2). This gain and loss explains why some homolog families are shared by distant lineages but not by closer ones. Nonetheless, it is important to consider that the new genomes are in draft form and are divided into contigs. Thus, these genomes contain fragmented CDSs that can artificially increase the number of predicted gene families and introduce some bias into subsequent analysis.

### Pairwise comparison of the genomic content of Moko with the NPB and brown rot strains

To overcome the problem of these mosaic genomes and to specifically target gene content associated with host adaptation, we hypothesized that the brown rot clade emerged when the IIB-1 brown rot strains diverged from the IIB-3 Moko lineage. Similarly, we hypothesized that the NPB clade emerged when the IIB-4 NPB lineage diverged from the IIB-4 Moko strains. This methodology thus focuses on the evolution of genomic content during emergence events. To test these hypotheses, we directly compared the co-occurrence patterns of genes of the IIB-1 brown rot genomes to those of the Moko genomes and the IIB-4 NPB genomes to the Moko genomes. The first step was to establish which genes were unique to the brown rot and NPB strains compared with the Moko strains. Subsequently, we determined which genes were conserved in every Moko lineage but were absent in either the NPB or brown rot strains (Additional file 3).

The NPB strains exhibited 102 unique genes missing from all Moko strains. These genes fell into three putative genomic islands. The first one encoded a type I secretion system (T1SS), including an outer membrane protein (RALP6v1_3180004), a membrane fusion protein (RALP6v1_3180005), and an ATP-binding protein (RALP6v1_3180006). This secretion system is located next to a transposase homolog (RALP6v1_3180003), suggesting that the genes were most likely acquired by HGT. The second genomic island contained genes related to the degradation of the plant alkaloid isoquinoline (*iorA* and *iorB*) located next to a gene encoding an AraC-type transcriptional regulator. The third genomic island contained a gene with a phosphatase 2C domain, a motif usually found in eukaryotic organisms. Phosphatase 2C can be involved in various cell functions, including the regulation of plant defense in Arabidopsis [22], and is sometimes secreted into host cells by pathogens [23]. The genomic island also contained a gene encoding a putative helix-turn-helix (HTH)-type transcriptional regulator as well as transposition-related genes, suggesting that it was also acquired by HGT. Outside of these clearly defined putative genomic islands, CDSs encoding another HTH-type transcriptional regulator and a GGDEF-domain signal transduction protein were unique to the NPB genomes.

Only six genes were conserved in Moko strains but absent from NPB strains. Interestingly, this gene-set included *ripAA,* a well-characterized T3e formerly known as *avrA* [8], which was either absent or pseudogenized in NPB strains. This effector was highly conserved in Moko strains (~85% nucleotide identity) and in the species complex (present in 15 out of the 19 sequenced strains), thus suggesting that *ripAA* was lost during the emergence of NPB. Another GGDEF-domain signal transduction protein was also absent from the NPB strains.

Brown rot strains exhibited 134 conserved genes that were absent from all Moko strains. One hundred three genes coded for conserved proteins of unknown function. However, a BLAST search on NCBI’s nr database revealed that several of these hypothetical proteins exhibited homologs in other genera of soil-dwelling and plant pathogenic bacteria (e.g., *Xanthomonas*, *Pseudomonas*, *Cupriavidus* and others). We identified a single putative functional genomic island related to iron metabolism: a FecR homolog (UW551v3_mp40033) located next to an iron transporter (UW551v3_mp40034) and a sigma 70 factor (UW551v3_mp40032). Another gene block coding for hypothetical proteins without significant BLAST hits was also located next to an AraC-type transcriptional regulator. Finally, a member of the RTX exotoxin family (UW551v4_570022) was unique to the brown rot strains.

We identified 63 genes that were conserved in Moko strains and missing from brown rot strains. A large gene block included several genes of interest: the T3e *ripAU*, a two-component system involved in the stress response of *cpxR* and *cpxA*, multiple drug efflux pumps from the Acr and RND families, and an AraC-type transcriptional regulator. Several isolated transcriptional regulators, including one each from the TetR and MarR families, were also identified.

Our analyses identified similar numbers of NPB- and brown rot-specific genes relative to Moko strains. Although NPB strains exhibited three specific genomic islands that may be related to adaptation (potentially acquired by HGT), brown rot strains exhibited more than one hundred specific genes encoding proteins of unknown function that appeared to be conserved in other bacteria. Although gene loss was globally more predominant in brown rot strains than in NPB strains, both ecotypes only lost a single distinct T3e each (*ripAU* and *ripAA,* respectively), possibly while diverging from their respective Moko lineages. These contrasting rates of gene loss between effectors relative to the rest of the genome suggest that the loss of these two effectors could have been key events in the emergence of these ecotypes.

### HGT events between Moko lineages

To test the hypothesis that adaptation to banana spread through Moko lineages of phylotype II via HGT, we searched for signs of transfer between Moko lineages using the AnGST algorithm. This algorithm compares gene trees with their associated species tree and infers both HGT events and the direction of transfer by identifying conflicts between the two. We only considered genes with HGT patterns involving all four Moko lineages and directions of transfers compatible with a unique original donor. According to our hypothesis, this unique donor would be pathogenic to banana (Additional file 2). This strategy identified nine genes. Five of these genes exhibited the IIA Moko lineages MRCA as the likely donor with the IIB Moko lineages as acceptors. Of these five genes, two encode the T3es *ripAD* and *ripG4* from the GALA family. The others encode two hypothetical proteins and a bacteriophage-related protein. Three genes exhibited the IIB-3 lineage as the donor and putatively encode a protein belonging to the beta-lactamase superfamily, the translation initiation factor *infA*, and *hrcT*, a key component of the T3SS. The only gene that apparently exhibited the IIB-4 lineage as a donor encodes a putative adenine-specific methyl-transferase.

This analysis suggests that a small minority of genes seems to have spread through every Moko lineage via a single lineage (the hypothetical original donor). Although the original donors were not fully consistent, more than half of the HGT events originally started from the IIA Moko’s MRCA and affected T3es that are likely to be directly involved in pathogenesis.

### Association of sequence polymorphisms with host-adaptation

Due to the phylogenetic proximity of NPB and brown rot strains to individual Moko lineages, we analyzed the diversity of their shared genomic content. We analyzed multiple alignments of protein sequences to search for non-synonymous single-nucleotide polymorphisms (NS-SNPs) and insertions and deletions (INDELs) associated with the host-adapted groups. We will refer to these features as host-adapted polymorphisms (HAPs). More precisely, we selected polymorphisms that exhibited the same form in Moko strains but a different form in either the NPB or brown rot strains (Additional file 4).

Of the 2,855 genes common to the NPB and Moko strains, 96% were polymorphic. However, only 24 genes (<1%) contained HAPs, including 16 within conserved domains in protein sequences. This result can be explained by the very low divergence of the IIB4-NPB and IIB4 Moko lineages, as only 14% of their common genes are polymorphic. Notable NS-SNPs were identified in the T3e *ripAN* and in a PadR-like transcription regulator involved in the response to phenolic compounds; notable INDELS were identified in *motB*, a flagellar motor protein involved in the response to chemotaxis signals, and *epsF*, a membrane protein involved in the secretion of EPS. NS-SNPs were also identified in two multidrug resistance proteins (MDRs) and an HTH-type transcriptional regulator.

Ninety-nine percent of the 2459 common genes present in both the brown rot and Moko strains were polymorphic. Of these, 1024 genes (42%) contained HAPs, including 742 within protein domains. Twenty T3es contained the majority of NS-SNPs, including five T3es with more than ten NS-SNPs (*ripV1*, *ripG6*, *ripC1*, *ripAO* and *ripAD*), three members of the GALA T3e family and *ripAB* and *ripAC*. Two regulators and subunits of the T3SS were polymorphic: *prhJ* and *hrpAFGHJ*. Several HAPs were found in genes encoding members of diverse metabolic pathways, notably the NorB and NasF enzymes of inorganic nitrogen metabolism. Finally, 22 putative transcriptional regulators of various families contained HAPs in their substrate-binding domains.

## Discussion

Using comparative genomic analysis, we compared the genomes of three host-adapted groups of strains from the phylotype II of *R. solanacearum*: brown rot strains adapted to potatoes and temperate climates, Moko strains adapted to bananas, and NPB strains adapted to melon or *Anthurium*. Together, our analyses demonstrated that the potato brown rot, Moko, and NPB strains constitute phylogenetically related populations that have adapted to biologically distinct host environmental conditions. Thus, these phylotype II groups, called ecotypes, provided a good model to explore host adaptation in *R. solanacearum*. We hypothesized that comparing the genomes of these closely related but biologically distinct groups could identify specific mechanisms of host adaptation.

Overall, the species complex appeared to be very dynamic, with a large pan-genome and a limited core genome. Specifically, the *R. solanacearum* T3e repertoire was unusually large compared with those of other well-known plant pathogens such as *Pseudomonas* spp. or *Xanthomonas* spp. [24,25]. The variability of T3es suggests that there may be extensive functional redundancy between effectors. Paralog families, such as the GALA effectors, are known to exhibit functional redundancies, and single effector mutations rarely produce a virulence defect [10]. These functional redundancies drastically reduce the analytic power of comparative genomics; therefore, more data about the specific virulence functions of individual T3es are needed to better understand the biological mechanisms underlying host adaptation. Such data are especially important considering that among the few functionally characterized T3es [26], only *ripR* (*popS*) is a member of the *R. solanacearum* core effectome.

Three competing hypotheses could explain the origin of polyphyly of the Moko strains. The first hypothesis proposes that the phylotype II MRCA was a banana pathogen and that the capacity to infect banana was subsequently lost in multiple clades after clonal expansion due to variations in environmental selection pressures. Our analyses did not identify any genes present in all Moko strains but absent in all other *R. solanacearum* strains. Although this observation seems to contradict our hypothesis, it does not invalidate the hypothesis but rather suggests that different parts of the set of hypothetical genes responsible for the banana virulent phenotype present in phylotype II’s MRCA could have been lost in non-Moko lineages. This phenomenon would have contributed to the mosaic nature of *R. solanacearum* genomes and would have been facilitated by functional redundancies.

Our second hypothesis states that banana pathogenicity emerged in a single clade and spread among phylotype II strains by HGT facilitated by natural transformation. Despite the large gene pool shared by Moko strains, only nine genes exhibited HGT patterns consistent with our hypothesis. We determined that the T3e *ripAD* that was transferred from the common ancestor of the IIA-6/24 Moko lineages to the IIB-3/4 Moko lineages. This effector does not possess the regulatory *hrp*_II_ box motif but is expressed in an *hrpB*-dependent manner and is translocated into plant cells [27]. Moreover, although its function is not characterized in *R. solanacearum,* it is also a member of the *hpx8* family found in plant pathogenic *Xanthomonas*. Another T3e, *ripG4*, exhibited similar HGT patterns originating from the IIA Moko lineages. This effector is a divergent member of the GALA family that interferes with PAMP-triggered immunity in *Arabidopsis* and contributes to host adaptation [10].

A genomic content analysis was designed to characterize the evolution of genomic content during the emergence of the NPB and brown rot clades and to identify the specific genes that could be responsible for host range differences. Our analysis revealed that few genes separate the Moko strains from the NPB and brown rot strains despite their biologically distinct phenotypes. One of these, *ripAA,* was lost by NPB strains’ MRCA during its divergence from the IIB-4 Moko lineage. This effector is recognized by tobacco, so it prevents phylotype I strain GMI1000 from infecting tobacco plants [8]. We speculate that *ripAA* could either be an effector that enables Moko strains to wilt banana or an avirulence factor that prevents pathogenicity in plants that are not part of the Moko strains’ host range but are part of the NPB strains’ host range. *ripAA* is a common effector in the species complex, but none of the strains with this effector are melon or *Anthurium* pathogens. Therefore, we propose that the loss of this effector by the NPB strains could have been a key event in this clade’s host change. Similarly, *ripAU* was lost by the brown rot strains during the divergence from the IIB3 Moko lineage. This effector is also present in all of the other strains of the species complex, and its loss might have been necessary for brown rot strains to adapt to temperate climates. The cold tolerance of brown rot strains is strictly dependent on an interaction with the host [28], and T3es may play key roles in this interaction. Although the function of *ripAU* in *R. solanacearum* is unknown, this effector belongs to the *hpx8* family. Hpx families are T3e families sharing protein sequence similarities defined by Mukaihara, and the *hpx8* family shares similarities with the effector XopV, which is found in various plant pathogenic *Xanthomonas* [27]. However, the function of XopV in *Xanthomonas* is not well characterized.

A complete T1SS was acquired by NPB strains during their divergence from banana-infecting sequevar 4 Moko strains, most likely via HGT. T1SSs are ubiquitous and versatile systems often involved in the secretion of various virulence factors into the extracellular medium, such as proteases, toxins, or quorum-sensing molecules that can promote plant invasion [29]. T1SSs can also play a defensive role to counteract plant defense responses by exporting antimicrobial compounds out of the cell. Numerous genes encoding conserved proteins of unknown function were acquired by brown rot strains during their divergence from sequevar 3 Moko strains. Homologs of most of these genes are present in other soil-borne and plant pathogenic bacteria, suggesting that they may play a role in adaptation to either environmental conditions or unique hosts. Further characterization of these proteins of unknown function is required to determine if they participate in brown rot strain adaptation to temperate climate. Homologs of the transporter and the sensor of the iron-sensing *fec* system and a sigma70 factor were also encoded by brown rot-specific genes. The *fec* system is responsible for iron uptake and is essential in many human pathogens and a few enteric plant pathogens [30,31]. However, all *R. solanacearum* strains sequenced to date have the *fur* system, which depends on another siderophore*.* It is possible that this *fec* system has a ligand specificity that is more efficient at sequestering iron in potato tubers or at low temperatures. Functional analyses could determine if this additional iron uptake system is involved in brown rot cold tolerance. Finally, we looked for polymorphisms (i.e., HAPs) that could affect the function of the genes shared between Moko and either NPB or brown rot strains. Polymorphisms are introduced by replication errors or intragenic recombination and can have a major impact on gene functionality. In plant pathogens, a single amino acid modification in TAL effectors can alter the host gene targeted by the effector [32]. Despite the genetic distances between Moko lineages and their proximity to the brown rot and NPB ecotypes, a high number of genes contained sites that were coincidentally fixed in the Moko strains but polymorphic in either NPB or brown rot strains, suggesting that these sites are functionally important and may be related to host adaptation.

NPB and Moko strains contained several HAPs in known virulence factors. The T3e *ripAN* had a single NS-SNP. Although RipAN is translocated into host cells [33], this effector’s function in *R. solanacearum* is unknown. Homologs of *ripAN* have not been identified in other plant pathogenic bacteria. One HAP was also found in the gene encoding the inner membrane protein EpsF, which is predicted to be responsible for the modification and/or export of the exopolysaccharide EPS I, a major bacterial wilt virulence factor. A six-amino acid insertion was identified in the C-terminal end of the NPB *epsF* gene. The extent of cross-talk between plants and EPS is not yet entirely understood, but it is known that different EPS proteins trigger different plant defense responses and that EPS modifications play a role in biofilm formation, immune evasion and virulence in EPS-producing bacteria [34,35].

Furthermore, we also found a HAP in a transcriptional regulator containing a PadR-like domain. PadR domains modulate the expression of virulence factors, MDR efflux pumps and responses to phenolic stress [36,37]. Interestingly, HAPs were also identified in two independent MDR transporters. Because plants produce antimicrobial phenolic compounds in response to pathogens, the PadR and MDR genes might constitute a network for stress response/resistance to plant defense mechanisms adapted to NPB-specific host range. Altogether, the 24 HAPs identified may be necessary for NPB strains to adapt to the environmental conditions encountered in new hosts.

The brown rot and Moko strains exhibited hundreds of genes with HAPSs in conserved protein domains, suggesting that *R. solanacearum* metabolism and virulence may function differently in brown rot strains. Remarkably, the list of HAP sites included central virulence regulators, T3SS regulators, T3SS machinery, and 19 T3es. T3es with HAPs notably included an effector with a nuclear localization signal (*ripAB*) and several functionally redundant effectors known to affect host range (*ripG2*, *ripG3*, and *ripG6*) [10,38]. Recent transcriptomic studies suggest that regulation of virulence factors during pathogenesis is far more complex than previous models proposed [39,40]. Given that brown rot’s adaptation to cold is dependent on interactions with a host, a large reorganization of core mechanisms may be required for *R. solanacearum* to cause disease in temperate climates.

## Conclusions

This extensive comparative genomics analysis identified relatively few differences in gene content between closely related *R. solanacearum* strains with contrasting biological characteristics. However, several T3e were associated with the Moko, NPB and brown rot ecotypes (Table 2). Most differences between strains involved HAPs of uncertain biological significance, although many HAPs were located in genes associated with bacterial wilt virulence. Our study did identify specific hypotheses concerning mechanisms of host adaptation in the *R. solanacearum* species complex. These hypotheses will be tested using functional genomics experiments such as gene swaps and deletions to determine whether these mechanisms play roles in host specificity. However, an important proportion of these candidate genes are related to regulatory function, suggesting that host range could evolve through changes in regulation. Small genomic differences could lead to drastically different expression profiles when the bacterium infects different hosts. Due to their unexpected overall genomic similarity and their clear-cut differences in host range, IIB-4 Moko and NPB form an elegant model for transcriptomic studies designed to identify differentially expressed genes associated with host specificity.

**Table 2:** **Candidate T3e for host specificity identified in Moko, NPB and brown rot ecotypes**

**Gene name**	**Alternative name**	**Characteristics**
*ripAU*		Absent from brown rot, present in Moko
*ripAA*	*avrA*	Absent from NPB, present in Moko
*ripG4*	*GALA4*	HGT from IIA Moko to IIB Moko lineages
*ripAD*		HGT from IIA Moko to IIB Moko lineages; HAPs between brown rot and Moko
*ripAB*	*popB*	HAPs between brown rot and Moko
*ripAC*	*popC*	HAPs between brown rot and Moko
*ripAD*		HAPs between brown rot and Moko
*ripAE*		HAPs between brown rot and Moko
*ripAI*		HAPs between brown rot and Moko
*ripAO*		HAPs between brown rot and Moko
*ripAP*		HAPs between brown rot and Moko
*ripAY*		HAPs between brown rot and Moko
*ripB*		HAPs between brown rot and Moko
*ripC1*		HAPs between brown rot and Moko
*ripD*	*avrPphD*	HAPs between brown rot and Moko
*ripE2*		HAPs between brown rot and Moko
*ripF1*		HAPs between brown rot and Moko
*ripG2*	*GALA2*	HAPs between brown rot and Moko
*ripG3*	*GALA3*	HAPs between brown rot and Moko
*ripG6*	*GALA6*	HAPs between brown rot and Moko
*ripH1*	*HLK1*	HAPs between brown rot and Moko
*ripH2*	*HLK2*	HAPs between brown rot and Moko
*ripV1*		HAPs between brown rot and Moko
*ripW*	*popW*	HAPs between brown rot and Moko
*ripAN*		HAPS between NPB and Moko

## Methods

### Genome sequencing and assembly

Total DNA was extracted using the phenol-chloroform method. Libraries were constructed using Nextera technology and sequenced on Illumina’s HiSeq-2000 using a 2 × 50-nt paired-end strategy. Reads were pre-processed using Trimmomatic [41]. First, adapter sequences and low-quality nucleotides occurring at 5’ and 3’ ends with a Phred quality score < 20 were trimmed. Second, reads shorter than half their initial lengths were discarded. The resulting reads were assembled using Velvet [42]. Manual editing of the annotations was performed for genes of interest using the MaGe web interface of the MicroScope platform [43]. The accession numbers are as follows: CFBP1416 [EMBL:PRJEB7434], CIP417 [EMBL:PRJEB7427], B50 [EMBL:PRJEB7421], Grenada 91 [EMBL:PRJEB7428], UW179 [EMBL:PRJEB7426], UW163 [EMBL:PRJEB7430], CFBP6783 [EMBL:PRJEB7432], and IBSBF1503 [EMBL:PRJEB7433]. Annotation files are provided in genbank format (Additional file 5).

### Pathogenicity assays

Pathogenicity was assessed on 3–4 fully expanded leaves of tomato cv. L390 (T10) (30 plants), potato cv. Désirée (30 plants), Cavendish banana cv. 902 (8 plants), melon cv. Amish (30 plants) and the ornamental plant Anthurium cv. Fire (4 plants). Plants were placed in a full containment security level growth chamber with a 12-h photoperiod, 28 ± 2°C (day) and 24 ± 2°C (night), and 90% relative humidity. Bacterial suspensions were prepared as described in Cellier & Prior 2010. Using the soil-soak method, each plant was inoculated with 5x10^9^ colony-forming units (CFU) after lightly damaging the roots with a scalpel. Strains were considered pathogenic if more than 50% of plants presented wilt symptoms within one month after inoculation. Each assay was repeated 2 times. We could not analyze the phenotype of strain Po82 because the authors of this published genome would not share their strains [44].

### Exploratory virulence dataset

The list of *R. solanacearum* virulence factors analyzed in this study was based on Remenant et al. [12]. Orthology relationships were determined for each strain by BLAST and synteny data using the web-based MaGe interface. Every gene annotation was then manually validated to ensure homogeneity of the start codon positions and to detect frameshifts and pseudogenization. T3es were annotated using the IANT “Ralstonia T3E” database [13]. T3es located on contig borders were considered as present to establish the core effectome.

### Species complex phylogeny

The species complex phylogeny was inferred by neighbor-joining using MUMi genomic distances [20]. MUMi values were computed from whole-genome comparisons conducted with MUMMer 3.0 [45].

### Phyletic profiles

Homology relationships across all genomes analyzed in this study were inferred using the Orthologous MAtrix algorithm (OMA) [46] with the following default criteria: alignment length > 60% of the minimum gene length and alignment score > 181 in Gonnet PAM matrix units. Phyletic profiles [47] were subsequently determined using R to identify co-occurrence patterns for specific genes in a given group of strains. The specific gene content was locally blasted on the NCBI nr database to identify an eventual source organism.

### Detection of gain, loss and HGT events

Amino acid sequences of each homolog family were aligned with Muscle [48], and a neighbor-joining phylogenetic tree supported by 1000 bootstrap replicates was computed with FastTree [49]. Gain, loss, and HGT events were inferred by reconciliation of the gene trees and the species complex tree topologies using the AnGST algorithm (Analyzer of Gene and Species Tree) [50]. This algorithm compares the topology of a gene tree with its associated species tree, which is generally defined as an explicit phylogenetic method within the scope of HGT detection. The algorithm identifies differences between the gene and the species trees and explains them (“reconciles”) according to a set of evolutionary events, including gain, loss, duplication or horizontal transfer, inferred with a parsimony-based model. Loci with > 95% nucleotide identity were discarded to avoid false positives caused by phylogenetic trees with insufficient resolution.

### Allelic variation analysis

Nucleic and protein sequences of each homolog family were aligned with Muscle [48]. The resulting alignments were trimmed on the 5’ ends and then screened for host-associated amino-acid polymorphisms using R. The strain MolK2 was used as a reference to assign the position of each polymorphism in the sequence. Functional domains containing polymorphisms were identified using the CDD database from NCBI.

### Availability of supporting data

The data sets supporting the results of this article are included within the article and its additional files. Sequence data are available at the EMBL nucleotide sequence database:

- CFBP1416 http://www.ebi.ac.uk/ena/data/view/PRJEB7434
- CIP417 http://www.ebi.ac.uk/ena/data/view/PRJEB7427
- B50 http://www.ebi.ac.uk/ena/data/view/PRJEB7421
- Grenada 91 http://www.ebi.ac.uk/ena/data/view/PRJEB7428
- UW179 http://www.ebi.ac.uk/ena/data/view/PRJEB7426
- UW163 http://www.ebi.ac.uk/ena/data/view/PRJEB7430
- CFBP6783 http://www.ebi.ac.uk/ena/data/view/PRJEB7432
- ISBSF1503 http://www.ebi.ac.uk/ena/data/view/PRJEB7433

## Additional files

Additional file 1:
**Subset of 227 virulence factors from the pan-genome.** These genes were manually selected and annotated. Red cells: absent gene. Orange cells: pseudogenized of frameshifted gene. Black: Fragmented genes due to sequencing gaps. Additional file 2:
**Results from the analysis with the AnGST algorithm.** Gene gain and loss events in the species complex and HGT patterns between Moko lineages. Black boxes indicate that the lineage is involved in the HGT. Additional file 3:
**Results of phyletic profiling.** List of genes present or absent in NPB or brown rot strains when compared to Moko strains Results of BLASTp in the NCBI nr database are provided. Additional file 4:
**Results from the analysis of HAPs. List of HAPs contained in genes shared by NPB or brown rot strains with Moko strains are provided.** Protein domains predicted from NCBI CDD database and containing HAPs are indicated. Additional file 5:
**Annotations files (.gb).** Annotations are formatted in genbank format.

## Abbreviations

AnGST: Analyzer of gene and species tree
CDS: Coding sequences
EPS: Exopolysaccharide
HAP: Host associated polymorphism
HGT: Horizontal gene transfer
HTH: Helix turn helix
INDEL: Insertion deletion
MDR: Multidrug resistant
MLST: Multilocus sequence typing
MRCA: Most recent common ancestor
MUMi: Maximum unique matches index
NPB: Not pathogenic to banana
NS-SNP: Non-synonymous polymorphism
OMA: Orthologous matrix
RSSC: *R. solanacearum* species complex
T1SS: Type I secretion system
T3e: Type III effector
T3SS: Type III secretion system
